# Glances at the Hospitals

**Published:** 1901-10-26

**Authors:** 


### GLANCES AT THE HOSPITALS.

### THE GRIMSBY HOSPITAL.

There was a balance against the hospital of £804, but this has been reduced to £551, and the committee express the hope that careful management will still further diminish it. The total number of patients treated during the year was 22,294, and of these 593 were in-patients. This nnmber included 405 surgical cases and 188 medical cases. In the children's ward 139 were treated during the year. The average stay in the hospital was 26·4 days. The medical and surgical reports are so meagre that no useful information can be extracted from them, and we trust that the recently-appointed house surgeon will be able to improve and amplify them.

### VICTORIA HOSPITAL FOR CONSUMPTION AT CRAIGLEITH, EDINBURGH.

The income was £2,180 and the expenditure was £2,259. The hospital now contains 23 beds, 7 having been added in 1899. The building occupies one of the most beautiful sites in the vicinity of Edinburgh, and is surrounded by a richly-wooded park of 15 acres sloping towards the south. The grounds are provided with every facility for treatment in the way of covered shelters, rotating screens, and graduated walks. The committee remind us that 60,000 people die of consumption every year in the United Kingdom, and that 7,000 of these annual deaths take place in Scotland. They very pertinently ask, "What are 23 beds in view of the vast figures which have been quoted?" And they tell us that the hospital might have been filled ten times over with deserving cases. As it is, much useful work has been done. During the six years of its existence upwards of 500 patients have been treated. Last year 117 were admitted, and a crowd of 100 victims of phthisis are ever knocking at the door. Most of these 100 have to wait six or eight months before their turns come, and then the disease has advanced too far; sometimes it has carried the patient off. The committee point out that for every £75 they get, they can receive four patients, and treat them for three months each; and that for £1,000 they can provide accomodation for eight cases. As a matter of fact there is no better way of carrying out the open-air treatment of phthisis than in wooden huts, and these can be provided and furnished for less than £100. That there are sufficient numbers of charitable people in England to provide whatever is necessary for the suffering poor we have not the least doubt, and that these people will come forward when they are convinced of the necessity of the provision we have also no doubt. For the richer people able to pay £5 5s. or so a week, sanatoria are springing up all over England, but there is a third class likely to be forgotten, although quite as deserving as those whom we may call the hospital poor. That is the class between the two we have spoken of—people who dislike the idea of accepting charitable aid, but who cannot command anything like £5 5s. a week. Hospitals for these are urgently wanted in various districts of the United Kingdom, and by some means or other they should be forthcoming, either as private speculations, or as institutions built and equipped by philanthropists, and subsequently self-supporting. In its Report for next year we hope the Victoria Hospital for Consumption will favour us with more extended statistics, not only as to the results of treatment and average duration of patients' residence, but as to the number of the staff and the exact inclusive cost of each patient, or of each bed occupied. These hospitals are comparatively new in the land, and details of every kind would prove most useful.

